# The Role of TGF-*β* Signaling Pathways in Cancer and Its Potential as a Therapeutic Target

**DOI:** 10.1155/2021/6675208

**Published:** 2021-07-22

**Authors:** Yun Yang, Wen-Long Ye, Ruo-Nan Zhang, Xiao-Shun He, Jing-Ru Wang, Yu-Xuan Liu, Yi Wang, Xue-Mei Yang, Yu-Juan Zhang, Wen-Juan Gan

**Affiliations:** ^1^Department of Pathology, Medical College of Soochow University, Soochow University, Suzhou 215123, China; ^2^Department of Pathology, The First Affiliated Hospital of Soochow University, Soochow University, Suzhou 215006, China; ^3^Department of Pathology, Dushu Lake Hospital Affiliated of Soochow University, Soochow University, Suzhou 215124, China

## Abstract

The transforming growth factor-*β* (TGF-*β*) signaling pathway mediates various biological functions, and its dysregulation is closely related to the occurrence of malignant tumors. However, the role of TGF-*β* signaling in tumorigenesis and development is complex and contradictory. On the one hand, TGF-*β* signaling can exert antitumor effects by inhibiting proliferation or inducing apoptosis of cancer cells. On the other hand, TGF-*β* signaling may mediate oncogene effects by promoting metastasis, angiogenesis, and immune escape. This review summarizes the recent findings on molecular mechanisms of TGF-*β* signaling. Specifically, this review evaluates TGF-*β*′s therapeutic potential as a target by the following perspectives: ligands, receptors, and downstream signaling. We hope this review can trigger new ideas to improve the current clinical strategies to treat tumors related to the TGF-*β* signaling pathway.

## 1. Introduction

**Figure 1:**
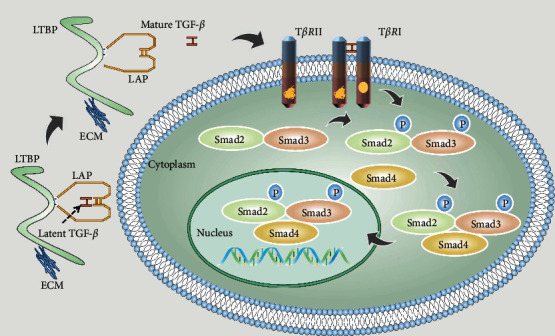
Mature TGF-*β* ligand, LAP, and LTBP together form a large latency complex that keeps the TGF-*β* ligand in a latent state at this time. When the TGF-*β* ligand is released from the complex, it changes from a latent to an active state. The released TGF-*β* ligand can directly bind to T*β*R II, thereby further binding to T*β*R I, but TGF-*β* ligand cannot directly bind to T*β*R I. The combination of TGF-*β* ligand, T*β*R II, and T*β*R I can further transmit signals to downstream mediators. After phosphorylation and activation of SMAD2 and SMAD3, they further bind to SMAD4 and transmit the signal to the nucleus.

The transforming growth factor-*β* (TGF-*β*) was first discovered in 1978 by JE de Larco and GJ Todaro in mouse fibroblasts transformed with murine sarcoma virus. TGF-*β* is a member of the cell growth factor superfamily; it is involved in the regulation of various biological processes, including cell growth, differentiation, autophagy, apoptosis, epithelial-mesenchymal transition (EMT), angiogenesis, inflammation, and immunity [1–5]. TGF-*β* mainly exerts multiple biological functions in the body through two pathways: the classic SMAD-dependent pathway and the non-SMAD-dependent pathway. In the SMAD-dependent classical pathway, there are two transmembrane Ser/Thr kinase receptors in the cell membrane, namely, TGF-*β* receptor I (T*β*R I) and TGF-*β* receptor II (T*β*R II). The combination of TGF-*β* and T*β*R II can activate the kinase activity of T*β*R I and induce the phosphorylation of T*β*R I. Subsequently, the activated T*β*R I can recruit and phosphorylate downstream SMAD proteins, SMAD2 and SMAD3. Once phosphorylated, SMAD2 and SMAD3 bind to the chaperone protein SMAD4 and are cotransported to the nucleus, where they can regulate the expression of TGF-*β* target genes [6, 7] (Figure 1). Here, we mainly discuss the related role of TGF-*β* in tumors and its potential as a therapeutic target. We first introduce the related role of this signaling pathway in tumorigenesis and development. Then, using the classic TGF-*β* signaling pathway as a framework, we discuss the molecules and mechanisms that cause the abnormal activation or inactivation of TGF-*β* from three perspectives. Finally, we summarize the current TGF-*β*-targeted tumor therapy drugs from these three perspectives. We hope that readers can expand the idea of designing new TGF-*β* tumor treatment drugs.

## 2. Introduction to the Related Mechanisms and Functions of TGF-*β* Signaling Pathway in Tumorigenesis and Development

**Figure 2:**
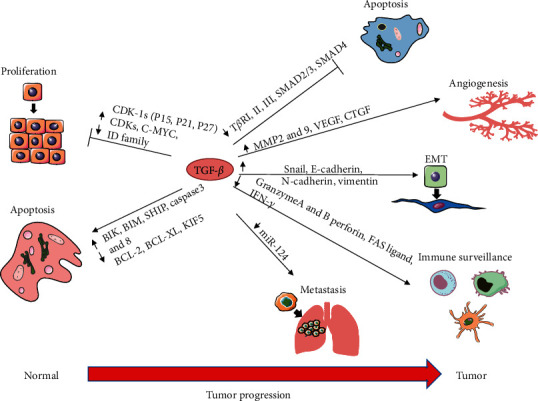
Dual effects of TGF-*β* on tumors. As a double-edged sword, TGF-*β* can promote and inhibit tumors through various mechanisms. TGF-*β* exerts its tumor inhibition mainly by inhibiting cell proliferation and inducing apoptosis. TGF-*β* can upregulate CDK-IS expression (P15, P21, and P27) to inhibit CDK and downregulate the expression of the C-MYC and ID family to inhibit cell proliferation. Simultaneously, TGF-*β* can also inhibit the expression of antiapoptotic genes such as BCL-X, BCL-2, and KIF5 and promote the expression of proapoptotic genes such as BIK, Caspase 3, and Caspase 8 to induce apoptosis and thus inhibit tumor growth. However, TGF-*β* can also promote cancer through several mechanisms. TGF-*β* can enhance EMT and metastasis to play its protumor role by upregulating Snail, E-cadherin, and N-cadherin or downregulating miR-124. It can also evade the immune system by inhibiting Granzyme AB, perforin, FAS ligands, and IFN-*γ* to achieve its tumor-promoting effects. TGF-*β* also triggers tumor growth by promoting angiogenesis by activating MMP2, MMP9, VEGF, and CTCT.

For the tumor, TGF-*β* is a double-edged sword, as it can achieve inhibition and promotion of tumors through various mechanisms (Figure 2). TGF-*β* has a strong cellular inhibitory ability and is a prominent antiproliferation agent. It can inhibit cell cycle progression by blocking the G1 phase and exert its antiproliferation ability by inhibiting proliferation drivers such as C-MYC and ID [8, 9]. TGF-*β* can also induce apoptosis to inhibit tumor growth [10]. In addition to acting directly on epithelial tumor cells, TGF-*β* can further control tumor development by regulating the production of growth factors in the surrounding stroma and the tumor microenvironment [8]. Furthermore, TGF-*β* inhibits inflammatory and immune processes [11]. However, when the immunosuppressive action of TGF-*β* becomes significant, it will eventually start promoting tumor progression. TGF-*β* inhibits the transcription of proapoptotic and lysogenic cytokines in cytotoxic T lymphocytes (CTLs), such as perforin, Granzyme A (GZMA), Granzyme B (GZMB), porphyrin interferon *g*

, and factor-associated suicide (FAS) ligands [12, 13]. TGF-*β* inhibits certain functions of CTLs, CD8^+^ T cells, and natural killer cells, resulting in a tumorigenic effect [14, 15]. TGF-*β* also enhances tumor invasiveness and angiogenesis by promoting the production and secretion of matrix metalloproteases proteinase-2 (MMP-2) and matrix metalloproteinase-2 (MMP-9) and downregulating the expression of tissue inhibitors of metalloproteases (TIMP) [8, 16–18]. TGF-*β* also induces EMT, which supports tumor invasion and spread by releasing tumor cells into the environment and promoting their movement [16].

### 2.1. Tumor Inhibition by TGF-*β*

#### 2.1.1. TGF-*β* Inhibits Tumors by Regulating Cell Proliferation

TGF-*β* inhibits cell proliferation primarily through two transcriptional events: the induction of cyclin-dependent kinase (CDK) inhibitors and C-MYC expression inhibition [19]. In neuronal, epithelial, and hematopoietic cells, TGF-*β* inhibits cell growth by targeting CDKs and their inhibitors (CDK-IS), responsible for controlling cell cycle progression beyond G1 during proliferation. P15INK4B, P21CIP1, and P27KIP1 are three CDK-IS whose expression is promoted by TGF-*β*, which also inhibits the cyclin-CDK complex, leading to cell cycle arrest of G1 phase [19–22]. P15 mainly blocks the interaction between CDK4/6 and cyclin D, thus inhibiting the cell cycle process in the late G1 phase [23]. As a CDK inhibitor, P27 can be removed from the cyclin D-CDK4 complex, then interact with the cyclin E-CDK2 complex, and inhibit the cyclin E-CDK2 complex. P21 can also inhibit the activity of the cyclin E-CDK2 complex [9, 23]. When these CDK complexes are inactive, retinoblastoma protein (pRb) phosphorylation is inhibited, and pRb phosphorylation is the main switch in cell cycle progression, thus preventing G1 cells from moving into the S phase. Simultaneously, TGF-*β* can downregulate C-MYC oncogene expression, thereby inhibiting cell proliferation. C-MYC allows cells to multiply indefinitely and promotes cell division. In epithelial cells, the TGF-*β*-induced SMAD complexes synergistically regulate C-MYC expression's downregulation with transcription factors P107, E2F4/E2F5, and CCAAT/enhancer-binding protein (C/EBP) [24]. In addition, TGF-*β* inhibits Id1, Id2, and Id3 expression, which are nuclear factors associated with the G1 to S cell cycle transition. Inhibition of Id family proteins by TGF-*β* leads to decreased proliferation [25, 26]. The SMAD-dependent pathway of TGF-*β* is also associated with antiproliferative responses [27]. For example, TGF-*β* inhibits P70 S6 kinase by protein phosphatase 2A (PP2A) and induces G1 phase cell cycle arrest [28].

#### 2.1.2. TGF-*β* Inhibits Tumors by Promoting Apoptosis

TGF-*β* can trigger apoptosis of various cell types to inhibit tumor growth; there are two main pathways: the SMAD-dependent pathway and the independent pathway. However, the molecular mechanisms are still less clear. The SMAD-dependent pathway involves proapoptotic proteins such as death-related protein kinases (DAPK), Src homology inositol phosphatase (SHIP), and TGF-*β* induced early gene 1(TIEG1). Among them, DAPK can promote the release of cytochrome C and mediate TGF-*β*-dependent cell apoptosis by associating SMADs with mitochondrial proapoptotic events [29]. SHIP inhibits the PI3K-Akt pathway leading to cell death before cell survival [30]. TIEG1 can induce oxidative stress and produce reactive oxygen species (ROS) [31, 32]. These all promote apoptosis and thus inhibit tumor growth, and the expression of these proapoptotic proteins is regulated by TGF-*β*-mediated SMAD signaling. The DPC4-induced SAPK/JNK signaling pathway is also involved in TGF-*β* signaling, which leads to apoptosis [33]. In the TGF-*β* independent pathway, TGF-*β*-mediated apoptosis is involved in the activation of caspase. TGF-*β* inhibits the expression of antiapoptotic genes such as BCL-2 family members, BCL-XL, and X-linked inhibitor of apoptosis (XLAP) and promotes the expression of some proapoptotic genes such as caspase 3, caspase 8, and Bcl-2-interacting killer (BIK) [34–36]. Death domain-associated protein (DAXX) is a protein associated with the FAS receptor and is associated with the apoptotic signal of TGF-*β*. DAXX is involved in TGF-*β*-mediated JNK activation, thereby mediating programmed cell death [37]. TGF-*β* has been reported to increase the expression of the death-related protein kinase DAPK in liver cancer cells and signal transduction factors 45*β* for growth stagnation and DNA damage (GADD45beta) in the liver cells [38]. In hepatocytes, TGF-*β* induces cell death by producing ROS [39]. The production of TGF-*β*-induced ROS promotes apoptosis by regulating various members of the BCL-2 family, such as BCL-2 modifying factor (BMF) and BCL-2 interacting mediator (BIM) [40]. In gastric cancer cell lines, TGF-*β* mediates physiological apoptosis of gastric epithelial cells by activating apoptotic molecules BIM and Caspase 9 [41]. In pancreatic ductal adenocarcinoma (PDAC), TGF-*β* inhibits the expression of major gastrointestinal spectrum regulator, Krüppel-like Factor 5 (KLF5). However, KLF5 and SRY-related hug box 4 (SOX4) have synergistic effects, and the inhibition of KLF5 promotes apoptosis in the SOX4 program [42]. The mechanism by which TGF-*β* promotes the apoptotic responses that inhibit the tumors remains to be investigated. Further insights could provide new strategies for tumor inhibition and treatment.

### 2.2. Tumor Promotion by TGF-*β*

#### 2.2.1. Tumor Promoter Role of TGF-*β* in EMT

The EMT of tumor cells is a crucial step in tumor metastasis. EMT is essential in wound healing, fibrosis, cancer progression, and embryonic development [43]. TGF-*β* induces EMT during average growth and development. TGF-*β*-induced EMT supports tumor invasion and spread by releasing tumor cells into the environment and promoting their movement. In many cancers, TGF-*β* induces EMT with the transcriptional regulation of E-cadherin, N-cadherin, Snail, and vimentin [44, 45]. TGF-*β* and adhesion-dependent signaling are required for stable expression of myofibroblast phenotypes to induce cytoskeletal recombination [46]. After EMT, epithelial cells lose their polarity, tight junctions, and adhesion between cells, thus gaining the ability to migrate. This phenotypic change leads to reduced intercellular adhesion and enhanced migration and invasion ability of tumor cells, thus promoting cancer metastasis [47]. In breast cancer, the developmental transcription factor SOX4 can mediate TGF-*β*-induced action and promote EMT, tumor progression, and metastasis in breast cancer [48]. Moreover, the expression of TGF-*β* can also induce double mouse minute 2 (MDM2) expression, which makes p53 unstable, leading to EMT and tumor progression [49]. In p53-mutated cancers, TGF-*β* induces the assembly of the mutant p53, p63 protein complex, and SMADs. In this ternary complex, the tumor-suppressive function of P63 is antagonized, and the inactivation of P63 enabled both the mutant p53 and TGF-*β* to initiate EMT [50]. In addition, various studies have shown that TGF-*β* is involved in the EMT of tumor cells and the invasion and metastasis of a tumor cell. TGF-*β* promotes prostate cancer migration by inducing stress fiber aggregation and cytoskeletal rearrangement through the cell division cycle 42 (Cdc42), Rho A, and SMAD proteins [51]. TGF-*β* also induces the expression of dedicated for cytokinesis 4 (DOCK4) protein through the SAMD pathway, enhancing the exudation of lung cancer tumor cells and increasing the motility metastasis of tumor cells [52]. In non-small-cell lung cancer, activation of the TGF-*β* pathway leads to a severe loss of *miR-124*, enhancing EMT and metastasis [53]. TGF-*β*-driven EMT gives cancer cell motility, metastasis, and progenitor cell-like characteristics, all of which enable TGF-*β* to play its tumor-promoting role.

#### 2.2.2. Tumor Promoter Role of TGF-*β* in Angiogenesis

During tumor growth, the vascular network's development is essential because the proliferation and metastasis of tumor cells require nutrition and oxygen, which requires more angiogenesis. The expression level of angiogenic factors also reflects the invasion ability of the tumor [54]. Endothelial cells (EC) play a crucial role in angiogenesis. EC showed higher cell proliferation, migration, and invasion during neovasculature, and TGF-*β* signaling complexly correlates with EC ability and activity [55]. TGF-*β* also induces proangiogenic growth factors, such as vascular endothelial growth factor (VEGF) and connective tissue growth factor (CTGF), through fibroblasts and epithelial cells. These factors directly stimulate EC to form capillaries and play an essential role in inducing and maintaining tumor angiogenesis, thus accelerating cancer progression [56, 57]. Simultaneously, TGF-*β* can induce endothelial migration, which is necessary for angiogenesis [58, 59]. In liver cancer, prostate cancer, and renal cell carcinoma, high plasma levels of TGF-*β* are also associated with increased tumor angiogenesis and poor prognosis in these cancers [60, 61]. In non-small-cell lung cancer, the higher level of TGF-*β* in the tumor microenvironment is associated with increased angiogenesis, tumor progression, and poor prognosis [62]. In human breast cancer, the high mRNA levels of TGF-*β* are associated with increased microvascular density and these parameters are related to patients' poor prognosis [63]. TGF-*β*/SMAD4 signaling can upregulate the expression of miR-29a, which can target phosphatase and tensin homolog (PTEN) and activate the AKT pathway, thereby promoting the generation of new blood vessels [64]. In addition to TGF-*β* ligand action, TGF-*β* receptors are also critical for angiogenesis. TGF-*β* can enhance the expression of MMP9 and promote the formation of new blood vessels through one of its type I receptors, ALK5. TGF-*β*-ALK5 signal transduction can enhance the angiogenesis and invasiveness of breast cancer cells and prostate cancer cells [65, 66].

#### 2.2.3. Tumor Promoter Role of TGF-*β* in Immunologic Surveillance

TGF-*β* plays a systemic immune role and can significantly inhibit tumor immune surveillance of the host. TGF-*β* inhibits cytotoxic T cells, dendritic cells, and natural killer (NK) cells and produces a proinflammatory environment [67]. Cytotoxic CD8^+^T cells produce many cytokines, including perforin, GZMA, GZMB, IFN-*γ*, and FASL, which induce apoptosis of cancer cells. However, TGF-*β* can inhibit the expression of these cytotoxic genes through SMADs and ATF1. The neutralization of TGF-*β* in vivo restores the expression of critical cytotoxic genes involved in tumor clearance, thereby promoting the removal of antigen-specific tumors in vivo. These all suggest that TGF-*β* directly targets cytotoxic T cells to play its prooncogenic role during tumor evade immune surveillance [13]. Dendritic cells (DCs) are antigen-presenting cells responsible for inducing adaptive T cell response, and their activity has essential significance in antitumor immunity [68, 69]. TGF-*β* upregulates the differentiation inhibitor of TGF-*β*, Id1, and the overexpression of Id1 downregulates key factors of DC differentiation, leading to systemic immunosuppression [70]. NK cells also play an essential role in immune surveillance by directly recognizing tumor cells and initiating cytotoxic reactions [71]. TGF-*β* inhibits NK cell activation by diminishing the production of IL-15 and downregulating its active receptor natural killer group 2, member D (NKG2D) [72, 73]. In human glioma, TGF-*β* reduces the expression of NKG2D in CD8^+^ T and NK cells and inhibits the expression of the MICA, which is the ligand of NKG2D [74]. In addition to lymphocytes, TGF-*β* also has significant effects on some myeloid cells, which mainly consist of two myeloid cell types, namely, tumor-associated macrophages (TAM) and tumor-associated neutrophils (TAN). There are two phenotypes of TAM. The classically activated M1 phenotype can inhibit tumor growth, while the nonclassically activated M2 phenotype can promote tumor growth. TGF-*β* primarily drives the differentiation of the M2 phenotype of macrophages. They produce many different cytokines, such as MMP9, C-X-C motif ligand 8 (CXCL8), and IL-10, which can induce tumor growth and development [75]. Like TAM, TAN also has two phenotypes: antitumor phenotype (N1) and tumorigenic phenotype (N2). It has been shown that in the presence of TGF-*β*, neutrophils develop into an N2 phenotype that is not cytotoxic to the tumor. In the N2 phenotype, TAN's ability to secrete antitumor cytokines and activate cytotoxic T cells decreases, contributing to tumor growth and immunosuppression [76].

## 3. Introduction to the Molecules and Mechanisms That Regulate the TGF-*β* Signaling Pathway

As mentioned above, TGF-*β* can inhibit tumor occurrence by inhibiting cell proliferation and promoting cell apoptosis and tumor invasion and metastasis by inducing EMT, inducing angiogenesis, and inhibiting immunity. The TGF-*β* signaling pathway is precisely regulated under normal physiological conditions. Therefore, once the TGF-*β* signaling pathway is abnormally activated or blocked, this balance will be struck, aiding in the development of tumors. Next, we discuss some molecules and mechanisms that can activate or inhibit the TGF-*β* signaling pathway. We divide the molecules and mechanisms that regulate the TGF-*β* signaling pathway into the following three perspectives.

### 3.1. Regulation of the TGF-*β* Signaling Pathway at the Levels of the Ligands

Most TGF-*β* ligands exist in a latent state in the body, and the latent TGF-*β* binds noncovalently to the C-terminal prodomain latency-related peptide (LAP) to form a small latency complex (SLC). This small complex can further bind to the incubation period TGF-*β* binding protein 1 (LTBP1), and the three form a large latent complex (LLC) [77]. While being part of this structure, TGF-*β* cannot bind to its receptor and cannot exert its biological activity. It can only be connected to the extracellular matrix's binding site through LTBP [78]; in other words, TGF-*β* is latent. The process of transforming TGF-*β* from a latent state to an active state is called ligand activation. The TGF-*β* ligand must undergo activation to exert its biological activity. This feature also makes the regulation of the TGF-*β* activation process a critical point in regulating the TGF-*β* signaling pathway from the perspective of the ligand. The TGF-*β* ligand in the large latency complex cannot bind to the corresponding receptor. Therefore, the TGF-*β* ligand must be released from the large latency complex to make TGF-*β* active. The TGF-*β* ligand can be removed and activated from the complex in the following four ways.

1. Exposure to specific physical or chemical conditions, such as heat shock, extreme pH changes, ionizing radiation, and physical shearing force, can promote the separation of large complexes and activate TGF-*β* ligands [79–84].
2. Activation by enzymatic forms, including many different types of proteases, such as aspartic, cysteine, metalloproteinases, serine proteases, and neuraminidase expressed on the viral particles' surface, can release TGF-*β* ligands by inducing conformational changes in the latent complex [81, 85–89].
3. Some factors mainly act on LAP to activate TGF-*β*, such as ROS, thrombospondin 1 (TSP1) and members of the *α*v integrin family (including *α*v*β*1, *α*v*β*3, *α*v*β*5, and *α*v*β*6); these substances can release the noncovalent binding between LAP and TGF-*β* by acting on LAP, thereby releasing mature TGF-*β* [90–92].
4. Some substances can act on LTBP to activate TGF-*β.* From the previous description of the latent state of TGF-*β*, we can see that to release TGF-*β* from the complex, one way is to act on LAP, and the other is to act on LTBP. Most substances promote the activation of TGF-*β* by working on LAP. The three pathways we mentioned above can be summarized as using LAP as the point of action to activate TGF-*β* and LTBP as the trigger of TGF-*β* activation is relatively rare. Still, bone morphogenetic protein 1- (BMP1-) like protease can activate TGF-*β* by directly cleaving LTBP1 [93]. The substances that regulate the TGF-*β* signaling pathway at the level of the ligands include the ligands that activate TGF-*β* and the ligands that can inhibit the activation of the latent state TGF-*β*, such as the LSKL peptide, which is a competitive antagonist. The LSKL peptide inhibits the activation of TGF-*β* by preventing the interaction of TSP1 with the LAP of potential TGF-*β* [94], such as Emilin1, a cysteine-rich secreted glycoprotein expressed in the vascular tree. Emilin1 can inhibit TGF-*β* signaling by specifically binding to TGF-*β* to prevent TGF-*β* maturation [95]. Another example is Cripto, a developmental cancer protein that can prevent the TGF-*β* ligand from binding to the receptor by binding to the TGF-*β* ligand, thereby inhibiting TGF-*β* signaling [96].

### 3.2. Regulation of the TGF-*β* Signaling Pathway at the Level of the Receptors

All TGF-*β* ligands can bind to and activate the heterologous cell surface complex of their receptors. These receptors can be divided into T*β*R I and T*β*R II based on sequence similarity. Both T*β*R I and T*β*R II contain a serine/threonine kinase active transmembrane receptor [97]; the receptor structure can be divided into the extracellular domain and a transmembrane intracytoplasmic domain. In the intracytoplasmic region of T*β*R I, a highly conserved GS region (an area rich in glycine and serine residues) is the active region of T*β*R I kinase. This region can be phosphorylated by T*β*R II [98]. Although T*β*R II and T*β*R I are structurally similar, T*β*R II does not have a GS region but a short tail rich in serine and threonine at the hydroxyl end of the intracytoplasmic part. The TGF-*β* ligand itself cannot bind to T*β*R I. It can only attract T*β*R I to the cell surface after binding to T*β*R II and promote the phosphorylation of T*β*R I through T*β*R II. This staged process is essential for the smooth transmission of TGF-*β* signals. Therefore, the regulation from the receptors' perspective is another vital entry point for regulating TGF-*β* signaling pathways. Here, according to the regulatory mechanism, the TGF-*β* signaling pathway regulation from the receptor's perspective is divided into the following two ways.

#### 3.2.1. Regulatory Pathways Associated with Posttranslational Modification of Receptors

As mentioned above, the combination of T*β*R II and T*β*R I can promote the phosphorylation of T*β*R I. Phosphorylation of T*β*R I is an essential basis for TGF-*β* signal transduction. Only phosphorylation of T*β*R I can further activate downstream signaling mediators of the TGF-*β* signaling pathway, SMAD2, and SMAD3. Therefore, substances that affect receptor phosphorylation can activate or inhibit the TGF-*β* signaling pathway. For example, protein phosphatase 1 (PP1) can dephosphorylate T*β*R I to inhibit TGF-*β* signaling, SMAD anchor for receptor activation protein (SARA) can recruit the catalytic subunit of PP1 to dephosphorylate the receptor to inhibit the TGF-*β* signaling pathway [99], and 12 kDa FK506-binding protein (FKBP12) can bind to the GS domain of T*β*R I, thereby inhibiting the phosphorylation of T*β*R I [100]. Interestingly, SMAD7 can act on two posttranslational modification regulatory pathways. It can dephosphorylate and inactivate the receptor, and it can also induce receptor degradation by recruiting an E3 ubiquitin ligase. Eventually, the receptor is inactivated by dephosphorylation and is degraded by ubiquitination [101]. In addition to phosphorylation, ubiquitination is also a common posttranslational modification of TGF-*β* receptors. Therefore, substances that affect receptor ubiquitination can also regulate the TGF-*β* signaling pathway. SMAD7, FKBP12, and neural precursor cell expressed developmentally downregulated 4-like (NEDD4-2) also degrade the receptor by promoting its ubiquitination [102]. In contrast, C-CBL, heat shock protein 90 (Hsp90), transforming growth factor-beta stimulated clone 22 (TSC-22), tumor necrosis factor receptor-associated factor 4 (TRAF4), ubiquitin-specific protease 4 (USP4), ubiquitin-specific protease 11 (USP11), ubiquitin-specific protease 15 (USP15), and UCH37 can stabilize the receptor by blocking the ubiquitination of the receptor [103–110], thereby activating the TGF-*β* signaling pathway.

#### 3.2.2. Other Regulations besides the Posttranslational Modification

Regarding the regulation of the TGF-*β* signaling pathway at the level of the receptor, in addition to the posttranslational modification of the receptor, there are some factors or proteins that can directly interact with the receptor, causing the receptor to degrade and block signal transduction or after binding to the receptor, thus preventing the binding between the receptor and receptor, the binding between the receptor and ligand, and the binding between the receptor and the downstream players. Among others, these factors include toll-interacting protein (TOLLIP), salt-inducible kinases (SIK), caveolin-1 (CAV-1), Dapper 2 (dvl-associated proteins), and protein interacting with c-kinase 1 (PICK1). Their binding to the TGF-*β* receptor promotes receptor degradation, thereby inhibiting the TGF-*β* signaling pathway [111–115]. Other examples are BMP and activin membrane-bound inhibitor (BAMBI), FKBP12, serine-threonine kinase receptor-associated protein (STRAP), C-SKI (a transcriptional corepressor of SMAD-dependent TGF-*β*signaling), DRAK2 (A serine/threonine kinase belonging to the death-associated protein kinase family), ventricular zone-expressed pH domain-containing 1 (VEPH1), and additional substances that can bind to TGF-*β* receptors, thereby interfering with the binding of receptors to factors required for normal signal pathway transduction [116–120]. Of course, in addition to these factors (or proteins) that bind to receptors that can antagonize TGF-*β* signaling, some factors (or proteins) can interact with receptors to promote signaling. For example, 14-3-3ɛ, 14-3-3ɛ is the first protein other than SMADs that has been confirmed to interact with TGF-*β* receptors and activate signal transduction. It can interact with T*β*R I to induce TGF-*β*-induced signal transduction [121, 122]. For instance, the B*α* subunit of protein phosphatase 2A, the B*α* regulatory subunit, can interact with the cytoplasmic domain of T*β*R I to promote signal transduction [123]. Another example is disintegrin and metalloproteinase 12 (ADAM12). ADAM12 can bind to T*β*R II and stabilize the receptor by controlling the localization of the TGF-*β* receptor to the early endosome, thereby enhancing TGF-*β* signaling [124].

### 3.3. Regulation of the TGF-*β* Signaling Pathway at the Level of the Downstream Signaling

SMADs are crucial downstream signaling mediators of the TGF-*β* signaling pathway. The primary function is to transmit TGF-*β* signals from the cell membrane to the nucleus, thereby regulating the corresponding target genes' transcription and expression. Based on functional differences in the classic TGF-*β* signaling pathways, the SMAD proteins can be divided into three types. The first type includes the receptor-regulated SMADs, SMAD2, and SMAD3, which can be activated by T*β*R I-induced phosphorylation. The second is the universal SMAD, that is, SMAD4. SMAD4 can interact with SMAD2 and SMAD3 to help both transmit signals to the nucleus. The third includes the inhibitory SMADs, SMAD6, and SMAD7. The inhibitory SMAD proteins can negatively regulate the TGF-*β* signaling pathway through various mechanisms of action. Regardless of the SMAD protein, its activation or inhibition can affect TGF-*β* signal transduction, which is of great significance for TGF-*β* signal transduction.

#### 3.3.1. Regulatory Pathways Related to Posttranslational Modification of SMADs Protein

The posttranslational modification of the SMADs protein is the same as the posttranslational modification of the receptor, i.e., phosphorylation and ubiquitination. SMAD2 and SMAD3 can be phosphorylated and be further activated by T*β*R I, which means that the phosphorylation of SMAD2 and SMAD3 is necessary for the smooth transmission of the TGF-*β* signaling pathway. Thus, some factors or proteins that regulate the phosphorylation of SMAD2 and SMAD3 may influence the TGF-*β* signaling pathway, such as liver fibrosis-associated lncRNA1 (lnc-LFAR1) and lysyl oxidase-like 1 (LOXL1) [125, 126], both of which bind to SMAD2 and SMAD3 and promote their phosphorylation in the cytoplasm to activate the TGF-*β* signaling pathway, thereby stimulating the development of liver fibrosis. Some substances can inhibit the phosphorylation of these two SMADs by interacting with SMAD2 and SMAD3, such as protein phosphatase, Mg^2+^/Mn^2+^-dependent 1A (PPM1A), protocadherin gamma-A9 (PCDHGA9), heat shock protein 70 (Hsp70), and calcium-sensitive receptor (CaSR), ultimately inhibiting the conduction of the TGF-*β* signaling pathway by inhibiting the phosphorylation of SMAD2 and SMAD3 [127–130]. The phosphorylation of SMAD2 and SMAD3 activates TGF-*β* signaling, whereas ubiquitination and degradation of SMAD2 and SMAD3 inhibit TGF-*β* signaling transduction. For example, AXIN, DREB, and EAR motif protein 1 (DEAR1) can inhibit the TGF-*β* signaling pathway's conduction by promoting the degradation of SMAD3 ubiquitination [131, 132]. In contrast, OTU domain, ubiquitin aldehyde binding 1 (OTUB1), B-cell lymphoma-3 (BCL-3), ubiquitin carboxyl-terminal hydrolase 1 (UCHL1), and UCHL5 contribute to the deubiquitination of SMAD2 or SMAD3, making them more stable and less easily degradable and promoting TGF-*β* signaling [133–136]. The regulation of the TGF-*β* signaling pathway from the perspective of downstream signaling mediators includes not only SMAD2 or SMAD3 but also the regulation of universal SMAD and inhibitory SMAD, such as the wild-type p53-induced phosphatase 1 (Wip1), which selectively binds SMAD4 and dephosphorylates it, thereby inhibiting TGF-*β* signaling [137]. Examples include ubiquitin-specific protease 10 (USP10), which can act on SMAD4 to make it deubiquitinated and stable, further promoting TGF-*β* signaling [138], and USP26, which promotes SMAD7 deubiquitination, thereby amplifying the inhibitory effect of SMAD7 and strengthening the inhibition of the TGF-*β* signaling pathway [139].

#### 3.3.2. Regulatory Mechanisms beyond Posttranslational Modifications

In this section, in addition to the regulation of downstream media through posttranslational modification, we discuss two forms of regulation that enhance or hinder the synergism between SMADs by binding to SMADs to affect the entry of SMADs into the nucleus.

By affecting SMAD proteins' entry into the nucleus, players such as miR-26a, IL-6 (interleukin-6), and HSP72 can block the downstream signaling events of TGF-*β* by inhibiting the nuclear translocation of phosphorylated SMAD proteins [140–142]. Another example is PCDHGA9, a member of the cadherin family that inhibits not only the phosphorylation of SMAD2/3 but also the nuclear translocation of pSMAD2/3, inhibiting downstream signaling events of TGF-*β* through a dual-action. Compared to the number of substances that inhibit nuclear translocation, relatively few substances promote the nuclear translocation of SMAD proteins. These include importin 7 and importin 8 (Imp7 and Imp8) and the mammalian orthologues of Mask, which enhance the TGF-*β* signaling pathway transmission by assisting the nuclear translocation of SMAD proteins [143].

Besides these substances that can regulate the nuclear translocation of SMAD proteins, many other substances regulate SMADs protein in various ways. Examples include hepatocyte growth factor-regulated tyrosine kinase substrate (Hrs/Hgs) and SARA, both of which can promote TGF-*β* signaling by activating SMAD2 and SMAD3. Another example is endosome-associated FYVE-domain protein (Endofin), which promotes TGF-*β* signaling by promoting the binding of SMAD4 to SMAD2. One final example is CXXC‐type zinc finger protein 5 (CXXC5), which associates with the SMAD2/3 inhibitor histone deacetylase HDAC1 and competes with HDAC1 to bind to SMAD2/3, thereby eliminating the inhibitory effect of HDAC1 on TGF-*β* signal transduction and ultimately promoting TGF-*β* signal transduction [144–147]. In addition, *miR-326*, SKI, or SNON (members of the protooncoprotein family) can negatively regulate TGF-*β* signal transduction by inhibiting SMAD2, SMAD3, or SMAD4 [148, 149]. Transmembrane prostate androgen-induced protein (TMEPAI) and its homologs C18 ORF1, and ERBB2/Her2 receptor-interacting protein (ERBIN), among others, can also inhibit signal transduction by competing with SARA for binding to SMAD2/3 [150–152]. Interestingly, some substances can have a dual effect on SMAD proteins, both inhibiting and activating them. As a member of the GTPase Rho family (Rac1), Rac1 can inhibit TGF-*β* induced growth inhibition by inhibiting SMAD3 and promoting SMAD2 to enhance TGF-*β*-induced cell migration [153]. Nevertheless, how Rac1 coordinates the regulation of SMAD2 and SMAD3 in different cells is unknown.

## 4. Therapeutic Targeting of TGF-*β* Signaling Pathway

In the previous part of this article, we divided the molecules and mechanisms that regulate the TGF-*β* signaling pathway into three perspectives: the ligand; the receptor; and the downstream conduction media. Similarly, here, we summarize these three perspectives and divide the molecular compounds that target TGF-*β* tumor therapy into three categories: (1) tumor therapy targeting TGF-*β* ligand; (2) tumor therapy targeting TGF-*β* receptor; (3) tumor therapy targeting the downstream mediator of TGF-*β* (Table 1).

### 4.1. Tumor Therapy Targeting TGF-*β* Ligand

Antisense oligonucleotides (ASO) are short strands of deoxyribonucleotide analogs that can be hybridized with complementary mRNA to cause mRNA degradation or interfere with mRNA maturation, thereby downregulating the target gene expression [154]. AP12009 (trabedersen) is a TGF-*β*2-specific ASO. Trabedersen inhibits the proliferation and migration of pancreatic cancer cells and reverses the immunosuppressive effect mediated by TGF-*β*2, thereby exerting its antitumor activity in vivo [155]. Clinical I/II studies have confirmed that using AP12009 can prolong patient survival time with malignant glioma [156]. These results indicate that AP12009 can become an effective treatment for malignant tumors. AP11014 is a specific ASO for TGF-*β*1. Ongoing preclinical research is studying the efficacy of AP11014 in non-small-cell lung cancer, colorectal cancer, and prostate cancer [157].

TGF-*β* is expressed in most cells as the latent form (L-TGF-*β*). TGF-*β* must be activated to exert its cell proliferation and invasion functions, immune regulation, and angiogenesis. The combination with integrin can activate TGF-*β* [158]. Therefore, blocking integrin-mediated TGF-*β* activation has also become a new strategy to target TGF-*β* signaling. In breast cancer models, the use of 264RAD, an antibody that blocks integrin *α*v*β*6, prevents tumor growth effectively [159]. However, in a trial using the antibody EMD121974 (cilengitide), which selectively inhibits *α*v*β*3 and *α*v*β*5 integrins, to treat head and neck squamous cell carcinoma (stages I and II, NCT00705016), cilengitide did not improve the median survival time of patients compared with standard chemotherapy [160]. Similarly, a phase III clinical trial of glioblastoma found that adding cilengitide to temozolomide chemoradiotherapy did not improve the treatment effect [161].

A monoclonal antibody is an effective tool to inhibit TGF-*β* signal transduction, which exerts antitumor activity in various tumor models by blocking TGF-*β* binding to its receptor. In the 4T1 syngeneic mouse model of metastatic breast cancer, the treatment of mice with 1D11 can significantly inhibit breast cancer's lung metastasis, related to the salivary bone protein (Bsp) in the metastasis [162]. The same researcher found that 1D11 can also inhibit lung metastasis in a mouse model of metastatic breast cancer by increasing CD8^+^ T cells [163]. Another monoclonal antibody, 2G7, also exhibited a similar effect on inhibiting breast cancer metastasis by increasing NK cells' activity [164]. GC1008 (fresolimumab) is a high-affinity human monoclonal antibody that can neutralize the three active forms of TGF-*β* (TGF-*β*1, TGF-*β*2, and TGF-*β*3). Phase I studies have shown that GC1008 has significant antitumor activity in patients with advanced malignant melanoma and renal cell carcinoma [165]. In patients with metastatic breast cancer, fresolimumab combined with radiotherapy can improve patient median survival, which may be related to the enhanced systemic immune response. XPA-42-068, XPA-42-681, and XPA-42-089 are all human monoclonal antibodies with a high affinity that can neutralize various TGF-*β* isoforms. In a xenograft model of pharyngeal carcinoma, these antibodies can inhibit tumor growth [166]. At present, the pan-neutralizing antibody NIS793, which can block the three isotypes of TGF-*β*, is being used in the phase I/Ib study of patients with advanced malignant tumors combined with PD-1 antibody (PDR001) (NCT02947165).

### 4.2. Tumor Therapy Targeting TGF-*β* Receptors

With the participation of T*β*R III, activated TGF-*β* binds to T*β*R II with high affinity to recruit T*β*R I to the TGF-*β*/T*β*R II complex, phosphorylate SMAD2 and SMAD3, and initiate the signal transduction pathway. The TGF-*β* receptor plays a vital role in this pathway. Therefore, research on TGF-*β* receptor kinase inhibitors has also become a hot spot. At present, many T*β*R I (ALK5) inhibitors have been developed, most of which target the kinase domain of T*β*R I, thereby affecting the TGF-*β* signal transduction pathway. In many preclinical experiments, T*β*R I inhibitors have shown significant antitumor activity. As a small molecule selective inhibitor of ALK-5, SB431542 inhibits TGF-*β*-induced cell proliferation and migration in human glioma cells. It inhibits myeloma growth by restoring the terminal osteogenesis cell differentiation in a myeloma mouse model [167, 168]. SB431542 also inhibits the vasculogenic mimicry (VM) formation in xenografts in mouse models of breast cancer and inhibits tumor growth [169]. This discovery provides a new strategy for breast cancer treatment. Other studies have shown that SB431542 blocks HCC cell proliferation mediated by TGF-*β* signaling in vivo and in vitro related to the decrease of KLF6 expression in HCC cells [170]. SB505124 is another T*β*R I inhibitor that inhibits the activation of fibroblasts induced by TGF-*β*, thereby preventing esophageal squamous cell carcinoma- (ESCC-) induced neoangiogenesis [171]. In the pancreatic ductal adenocarcinoma mouse model, SB505124 significantly reduces pancreatic cancer cell proliferation, tumor growth, and metastasis [172]. SD208 is an oral T*β*R I inhibitor. In mouse models of pancreatic cancer and melanoma, SD208 inhibits pancreatic adenocarcinoma progression and reduces the development of melanoma bone metastasis and osteolytic lesions [173, 174]. In SW-48 colon adenocarcinoma cells, SD208 can also significantly downregulate the expression of oncogene miR-135b and reduce the occurrence of colon tumors [175]. LY2109761 is a small-molecule inhibitor that has stable pharmacokinetic characteristics that can inhibit T*β*R I and T*β*R II dually. In the mouse colon cancer cell CT26, LY2109761 reduces TGF-*β*-mediated cell migration and invasion. In vivo experiments have found that LY2109761 can reduce colon cancer liver metastasis and prolong the survival period of mice [176]. Similarly, in animal models of pancreatic cancer, LY2109761 inhibits abdominal organ metastasis of pancreatic cancer, especially liver metastasis, and improves its mortality [177]. In HCC cells, LY2109761 prevents HCC cell migration and invasion by upregulating E-cadherin [178] and can also exert antitumor activity by inhibiting HCC neoangiogenesis [179]. LY2109761 can also reduce the migration and invasion of glioblastoma cells and inhibit new vessel formation [180]. Galunisertib (LY2157299 monohydrate) is an oral small-molecule inhibitor (SMI) of T*β*R I kinase, which can block the conduction of the TGF-*β*/ALK5 signaling pathway by downregulating the level of SMAD2 phosphorylation [181]. Studies have found that galunisertib inhibits TGF-*β*1-mediated EMT and tumor cell migration, reverses TGF-*β*1-mediated CD8^+^ T cell and NK cell immunosuppression, and exerts a potent antitumor effect in various tumor models (including MX1 human xenograft breast cancer model, Calu6 human xenograft lung cancer model, and 4T1 breast tumor model) [182]. Phase II clinical trials have shown that galunisertib, combined with gemcitabine, can prolong patient median survival with unresectable pancreatic cancer and good safety [183]. Similarly, in a clinical phase IB study conducted in Japan, galunisertib, along with sorafenib, showed good safety and tolerability for treating patients with unresectable hepatocellular carcinoma [184]. However, in the phase II trial of recurrent glioblastoma, compared with lomustine plus placebo, the combined treatment of nilotinib and lomustine did not significantly improve patient overall survival [185]. At present, the second-generation ALK5 inhibitor LY3200882 has been developed. Compared with the LY2157299 compound, LY3200882 is more specific and potent. However, it is still in phase I clinical trial for treating patients with solid tumors, and its safety needs to be further verified (NCT02937272).

Many new T*β*R I small-molecule inhibitors have been developed recently, such as EW-7203 and EW-7195. These small-molecule inhibitors reduce the phosphorylation level of SMAD2 in vivo effectively and inhibit SMAD signaling and EMT induced by TGF-*β*1. In a mouse model of xenograft breast cancer, both EW-7203 and EW-7195 inhibit breast cancer lung metastasis [186, 187]. The study has found that compared with treatment with tyrosine kinase inhibitor (TKI) alone, the combined use of EW-7197 and TKI can delay the recurrence of the disease significantly in chronic myeloid leukemia (CML) mice, increase their survival period, and eliminate CML leukemia-initiating cells effectively [188]. Phase I/II clinical trials of EW-7197 (vactosertib) combined with other drugs for treating malignant tumors are currently underway, including metastatic gastric cancer (VAC + paclitaxel), advanced NSCLC (VAC + durvalumab), metastatic colorectal cancer, and gastric cancer (VAC + pembrolizumab), and progressive glioma (VAC + imatinib) [11]. The use of T*β*RI inhibitors inhibits most TGF-*β* signal transduction, but these kinase inhibitors usually lack specificity. At present, researchers have developed a small-molecule inhibitor CJJ300 that targets T*β*R II, which disrupts the formation of TGF-*β*-T*β*R I-T*β*R II signaling complex to inhibit the phosphorylation of SMAD and EMT induced by TGF-*β*. It is a novel mechanism to inhibit TGF-*β* signaling [189].

A new type of immunotherapeutic strategy has recently been developed for targeted TGF-*β* signal transduction, the bifunctional antibody-ligand trap. The antibody trap combines an antibody targeting CTLA-4 or PD-L1 and then fuses with the extracellular domain sequence of T*β*R II to disable TGF-*β* in the tumor microenvironment. Compared with standard anti-CTLA4 monotherapy, anti-CTLA4-T*β*R II molecules show more robust antitumor activity in human melanoma mouse models [190]. M7824 is an anti-PD-L1/T*β*R II fusion protein. Preclinical studies have found that M7824 can inhibit the EMT induced by TGF-*β* and exert antitumor activity in various antitumor models [191]. Phase I studies have shown that M7824 has good antitumor activity in patients with advanced solid tumors and has stable safety [192]. Several phase II/III trials are underway to evaluate the effect of M7824 for treating malignant tumors currently, such as metastatic colorectal cancer or advanced solid tumors with microsatellite instability, locally advanced or metastatic biliary tract cancer, and advanced non-small-cell lung cancer (NCT03436563; NCT03833661; NCT03631706).

### 4.3. Tumor Therapy Targeting TGF-*β* Downstream Transducers

As an important downstream mediator of the TGF-*β* signaling pathway, the SMAD protein plays an important role in TGF-*β* signaling. Therefore, interference with SMAD expression will also affect TGF-*β* signaling. The peptide aptamer is a good example. It is a small-molecule protein that can bind to protein targets [193]. According to research reports, in NMuMG mouse mammary epithelial cells expressing the peptide aptamer Trx-SARA, Trx-SARA binds explicitly to SMAD2 and SMAD3, reducing the level of SMAD2-SMAD4 and SMAD3-SMAD4 complexes while inhibiting TGF-*β*-induced EMT [194]. Recently, a small-molecule peptide TAT-SNX9 that can specifically target phosphorylated SMAD3 (pSMAD3) was discovered. In a mouse lung fibrosis model, TAT-SNX9 inhibits TGF-*β*-mediated fibers by targeting pSMAD3 [195].

## 5. Conclusions and Perspectives

The members of the TGF-*β* family are highly conserved cell signaling proteins with multiple functions. They play an irreplaceable role in human body homeostasis by regulating cell proliferation, movement, differentiation, and apoptosis. The role of TGF-*β* in tumorigenesis and development is complex and contradictory. In the early stage of cancer, TGF-*β* suppresses cancer by inducing cell cycle arrest and apoptosis. However, in the later stages of cancer, TGF-*β* turns into a tumor promoter, which induces EMT and angiogenesis and inhibits immune cell activity, thereby evading immune surveillance and promoting tumor growth and invasion. TGF-*β* signaling is under fine regulation in the body. Once abnormally activated or inactivated, it may break the body's homeostasis, cause dynamic imbalance, and further promote tumor occurrence and development. Although researchers are developing or have developed many tumoricidal agents targeting TGF-*β*, the ideal clinical application of TGF-*β* targeted therapeutic drugs in oncology has not been achieved. Is this related to the dual, contradictory role of TGF-*β* in the tumor? Might some TGF-*β*-targeted antagonists not only inhibit the tumor-promoting effect of TGF-*β* but also inhibit the tumor inhibitory effect of TGF-*β*. Or is it related to cell specificity and tissue characteristics? Different tissues, different cells, and different tumor microenvironments have different responses to different antagonists. Some factors activate TGF-*β* in a certain tissue but may not or even have a negative effect on TGF-*β* in other tissues. This may also be a new challenge for developing TGF-*β* targeted therapy drugs. In the future, how should we make full use of the anticancer effect of TGF-*β*? Should we avoid or even limit the role of TGF-*β* in promoting cancer? How to combine cell specificity and tissue specificity to restrict the use of TGF-*β* targeted drugs in different diseases? These questions require further exploration and discovery.

## Figures and Tables

**Table 1:** Summary of targeted TGF-*β* drugs.

Therapy	Target	Drug	Phase
Targeting TGF-*β* ligand	TGF-*β*2mRNA	AP12009 (trabedersen)	I/II/IIb
	TGF-*β*1mRNA	AP11014	Preclinical
	TGF-*β*1, *β*2, *β*3	1D11	Preclinical
		2G7	Preclinical
		XPA-42-068, XPA-42-681	Preclinical
		GC1008 (fresolimumab)	I/II
		NIS793	I/Ib
	TGF-*β*1, *β*2	XPA-42-089	Preclinical
	*α*v*β*6 Integrins	264RAD	Preclinical
	*α*v*β*3, *α*v*β*5 Integrins	EMD121974 (cilengitide)	I/II/III

Targeting TGF-*β* receptor	T*β*R I	SB431542	Preclinical
		SB505124	Preclinical
		SD208	Preclinical
		LY2157299 (galunisertib)	I/II
		LY3200882	I
		EW-7203, EW-7195	Preclinical
		EW-7197	I/II
	T*β*R I/II	LY2109761	Preclinical
	T*β*R II	CJJ300	Preclinical
	Chimeric antibody-TGF-*β* traps	CTLA4-T*β*R II	Preclinical
		PDL1-T*β*R II (M7824)	I/Ib/II/III

Targeting the downstream mediator of TGF-*β*	Smads	Trx-SARA	Preclinical
	pSmad3	TAT-SNX9	Preclinical

## Data Availability

The datasets used and analyzed during the current study are available from the corresponding author upon reasonable request.
